# Male and female inheritance patterns in tetraploid ‘Moncada’ mandarin

**DOI:** 10.1007/s00299-019-02494-y

**Published:** 2019-11-28

**Authors:** Miguel Garavello, José Cuenca, Andrés Garcia-Lor, Neus Ortega, Luis Navarro, Patrick Ollitrault, Pablo Aleza

**Affiliations:** 1 Centro de Citricultura y Producción Vegetal, Instituto Valenciano de Investigaciones Agrarias (IVIA), Carretera CV-315, km 10.7, Moncada, 46113 Valencia, Spain; 2 INTA, Concordia Agricultural Experiment Station, 3200 Concordia, CC 34, Entre Ríos, Argentina; 3 Unité Mixte de Recherche, Amélioration Génétique et Adaptation des Plantes (UMR Agap), Centre de Coopération Internationale en Recherche Agronomique pour le Développement (CIRAD), Corse, 20230 San Giuliano, France

**Keywords:** Citrus, Triploid, Tetraploid, SSR and SNP markers, Disomic inheritance, Tetrasomic inheritance

## Abstract

**Key message:**

Tetraploid `Moncada´ mandarin, used as male and female in interploidy hybridizations, displays mainly tetrasomic inheritance for most LGs, with slight variations according to the direction of the crossing.

**Abstract:**

Triploid-breeding programs in citrus are key tool to develop seedless cultivars. Obtaining triploid citrus hybrids may be achieved through different strategies, such as the exploitation of female unreduced gamete in crosses between diploid parents and diploid by tetraploid sexual hybridizations, in which tetraploid genotypes can be used as male or female parents. Genetic configuration of triploid populations from interploid crosses greatly depends on the chromosomic segregation mode of the tetraploid parent used. Here, we have analyzed the inheritance of the tetraploid ‘Moncada’ mandarin and compared the genetic structures of the resulting gametes when used as male and as female parent. The preferential chromosome pairing rate is calculated from the parental heterozygosity restitution (PHR) of codominant molecular markers, indicating the proportion between disomic and tetrasomic segregation. Tetraploid ‘Moncada’ both as female and male parent largely exhibited tetrasomic segregation. However, as female parent, one linkage group (LG8) showed intermediate segregation with tendency towards tetrasomic inheritance, while another linkage group (LG4) evidenced a clear intermediate segregation. On the other hand, when used as male parent two linkage groups (LG5 and LG6) showed values that fit an intermediate inheritance model with tetrasomic tendency. Significant doubled reduction (DR) rates were observed in five linkage groups as female parent, and in six linkage groups as male parent. The new knowledge generated here will serve to define crossing strategies in citrus improvement programs to efficiently obtain new varieties of interest in the global fresh consumption market.

**Electronic supplementary material:**

The online version of this article (10.1007/s00299-019-02494-y) contains supplementary material, which is available to authorized users.

## Introduction

Polyploids are plants with somatic cells that contain three or more complete sets of chromosomes (Ramsey and Schemske 1998). Ancient whole-genome duplications have been reported in most evolutionary lineages and may represent a crucial mode of speciation and eukaryotic genome evolution (Cai et al. 2019; Van de Peer et al. 2017). In fact, all the angiosperm genomes sequenced to date exhibit evidence of ancient polyploidization events (Cai et al. 2019; Soltis et al. 2014; Van de Peer et al. 2017) and polyploidy is one of the major forces of evolution for plant species, leading to their diversification and differentiation (Gallais 2003; Otto and Whitton 2000; Van de Peer et al. 2017).

Basically, polyploids differ from the diploid counterparts in their ecological, morphological, and physiological characteristics (Dewitte et al. 2009; Guerra et al. 2014; Ramsey 2007; Ruiz et al. 2016). Several mechanisms lead to polyploidy, such as somatic doubling or the production of unreduced gametes which is the main polyploidization mechanism reported in plants (Bretagnolle and Thompson 1995; De Storme and Geelen 2013; Ramsey and Schemske 1998, 2002).

Polyploidization offers many opportunities as a valuable tool in citrus-breeding programs (Aleza et al. 2016; Cuenca et al. 2015; Grosser and Gmitter 2011; Ollitrault et al. 2008). In *Citrus* and related genera, diploid genotypes are the most common, with a basic chromosome number *x* = 9 (Krug 1943). However, euploids and aneuploids have been induced or found occasionally, with triploids and tetraploids being the most common euploid variations (Lee 1988). Citrus triploid genotypes are generally seedless, a demanded characteristic for fresh fruit marketing (Aleza et al. 2012a, b, 2016). However, a few seedy triploid lime varieties have been described (Curk et al. 2016). Triploid genotypes in citrus are routinely obtained by sexual hybridization, through unreduced female gametes (Aleza et al. 2016; Cuenca et al. 2011, 2015), and interploid hybridizations between diploid and tetraploid genotypes (Aleza et al. 2012a, b; Grosser and Gmitter 2011; Starrantino and Recupero 1982).

There are two extreme models for diploid gametes produced by tetraploid plants, i.e., disomic in allotetraploids and tetrasomic in autotetraploids (Stebbins 1947; Stift et al. 2008; Sybenga 2012). The fusion of the genomes of two species gives rise to the allotetraploids, which present two sets of homologous chromosomes. During meiosis, each chromosome is paired with its homologous and forms only bivalents (Stebbins 1947; Sybenga 2012). This generates a 100% interspecific heterozygosity transmitted by each gamete, resulting in a disomic inheritance (Stift et al. 2008). In contrast, the four homologous chromosomes in the autotetraploids have the same opportunity to mate during meiosis, leading to multivalent formation and thus, tetrasomic inheritance (Aleza et al. 2016; Jackson and Jackson 1996; Sybenga 1996). For autotetraploids resulting from somatic chromosome doubling of diploid varieties, it theoretically leads to 66% restitution of the heterozygosity of the diploid that originates the tetraploid (Aleza et al. 2016; Sanford 1983). In fact, allo- and autotetraploids are the extremes of the range. In cases where parents are divergent, but have retained enough homology to prevent exclusive preferential pairing, inheritance patterns intermediate between di- and tetrasomic can be expected (Jeridi et al. 2012; Stebbins 1947; Stift et al. 2008; Sybenga 1996). Intermediate inheritance patterns have been revealed in citrus allotetraploid somatic hybrids (Kamiri et al. 2011, 2018) and for the tetraploid ‘Clemenules’ clementine (Aleza et al. 2016). Stift et al. (2008) developed a likelihood-based approach to evaluate whether disomic, intermediate, or tetrasomic inheritances best fitted the segregation of genetic markers and to estimate preferential pairing and double reduction (DR) rates. DRs can occur when tetravalent are formed and increase the homozygosity of diploid gametes (Aleza et al. 2016; Ronfort et al. 1998; Stift et al. 2008; Sybenga 1996). A simplified likelihood method was proposed by Aleza et al. (2016) for tetraploid resulting from somatic chromosome doubling.

Molecular marker analysis indicate that cultivated citrus resulted from complex interspecific admixtures of four ancestral taxa: *C. reticulata* (mandarin), *C. maxima* (pummelo), *C. medica* (citron), and *C. micrantha* (papeda) that arose during the domestication of citrus fruits (Curk et al. 2016; Froelicher et al. 2011; Garcia-Lor et al. 2013b; Nicolosi et al. 2000) and these results were confirmed by sequencing data (Wu et al. 2014, 2018; Xu et al. 2013). Commonly, the tetraploid parents used in interploid hybridizations for triploid breeding result from somatic chromosome doubling occurring spontaneously in nucellar cells or induced by treatment using antimitotic agents such as colchicine and oryzaline (Aleza et al. 2009, 2011). In relation with the phylogenetic origin of the parental diploid such somatic tetraploids can be autotetraploid for monospecific varieties, allotetraploids when parental diploid resulted from direct interspecific hybridization or segmental allotetraploid when parental diploid had a more complex admixture genome. These complex genomes may, therefore, impact the observed segregations in breeding programs.

Here, we analyze the segregation pattern of the tetraploid ‘Moncada’ mandarin used both as male and as female parent in interploid crosses by genotyping triploid progenies with Simple Sequence Repeat (SSR) and Single-Nucleotide Polymorphism (SNP) molecular markers.

Diploid ‘Moncada’ mandarin was obtained from after 1980 in a breeding program held at Instituto Valenciano de Investigaciones Agrarias (IVIA) from a handmade pollination between ‘Oroval’ clementine (*Citrus clementina* Hort. Ex Tan.) and ‘Kara’ mandarin (*C. unshiu* (Mak) Marc. × *C. nobilis* Lour.) (Bermejo et al. 2011). Later, tetraploid ‘Moncada’ mandarin was obtained by colchicine treatment of shoot tips grafted in vitro (Aleza et al. 2009). This mandarin hybrid is characterized by its excellent fruit quality, very easy to peel, very late maturity period and also is a non-apomictic genotype what makes a very interesting parent in citrus-breeding programs based on sexual hybridizations aimed to recover large populations of triploid hybrids. The breeding implications of the use of the tetraploid ‘Moncada’ mandarin as male or female parent in the recovery of large populations of triploid hybrids are further discussed.

## Materials and methods

### Plant material

Triploid hybrid progenies were obtained from 4 × × 2× and 2× ×  4× sexual hybridizations using tetraploid ‘Moncada’ mandarin as female and male parent, respectively. Tetraploid ‘Moncada’ mandarin was obtained directly from shoot tip grafting combined with colchicine treatment (Aleza et al. 2009). In 4 × × 2× sexual hybridization, 72 triploid hybrids were recovered using diploid ‘Anana’ mandarin (*C. reticulata*) as male parent (from here on referred as MA hybridization), whereas in the 2 × × 4× sexual hybridization, 88 triploid hybrids were obtained with the non-apomictic diploid ‘Clemenules’ clementine female parent (from here on referred as CM hybridization). Ploidy-level analysis by flow cytometry and triploid hybrid recovery was performed following the methodology described by Aleza et al. (2012a, b).

### Genotyping of the triploid progenies

To study the genetic structure of the diploid gametes produced by the tetraploid ‘Moncada’ mandarin, progenies along with the parents were genotyped using SSR and SNP markers distributed homogeneously in the nine linkage groups (LGs) of the clementine reference genetic map (Ollitrault et al. 2012a). These markers were heterozygous for ‘Moncada’ mandarin and displayed polymorphism between ‘Moncada’ mandarin and ‘Clemenules’ or ‘Anana’ mandarins. Since ‘Moncada’ is a direct hybrid between clementine and ‘Kara’ mandarin, it was difficult to find heterozygous markers for ‘Moncada’ mandarin with polymorphism with clementine. Finally, 24 SSRs and 19 SNPs markers previously developed were analyzed for both populations. In addition, 11 new SNP markers were developed (Table 1) from a Genotyping-by-Sequencing (GBS) diversity analysis (unpublished data). Detailed information about SSR and SNP markers used in this study is given in Table 2. Given the genetic proximity between the tetraploid ‘Moncada’ and clementines, the exact same set of molecular markers could not be used in both families (CM and MA). Even so, 13 molecular markers were used in common for both families, distributed in eight out of the nine LGs.

**Table 1:** Primer sequences of the new SNP markers developed in this paper for use in KASPar™ assay

Markers name	SNP-specific primer		Common primer
C1P26815936	Allele X:	ATGATTGTCTCAGATACTGTTGAAGCT	AAAGCTGAGCTAGTTTCCCACTTTCATA
	Allele Y:	ATGATTGTCTCAGATACTGTTGAAGCA	
C2_23768463	Allele X:	CAAAGAACCCTCTTGCAGCGTG	CGTGCTTATACCTCTCCCATTGGTT
	Allele Y:	CAAAGAACCCTCTTGCAGCGTC	
C3_11509117	Allele X:	CAGAAGCCAAACCCACTTGATTTTC	AGTTTGCAGCTTTTGGGTGGGGAT
	Allele Y:	CAGAAGCCAAACCCACTTGATTTTG	
C4P229604	Allele X:	AGGATCTAATGCTATTGAGGACCTG	GTGCCCTTCAGGTTGATTAGAATTTGTTT
	Allele Y:	AAGGATCTAATGCTATTGAGGACCTA	
C4P25377913	Allele X:	AGTGTTTTACATAGTTCCCCTTTGGA	CACAAAAGGACCTGCAAATAGGAGTAAAA
	Allele Y:	GTGTTTTACATAGTTCCCCTTTGGG	
C4P5278891	Allele X:	GAATTACTGCAGCAACTTGAGAAGCA	ATAACGAGCTGTGCGTAGCCCATTA
	Allele Y:	AATTACTGCAGCAACTTGAGAAGCG	
C6_15847634	Allele X:	CGTTCAGGTGCACTGGCATTG	GCGAACGACTCAAGAATGCCTAGAA
	Allele Y:	CCGTTCAGGTGCACTGGCATTT	
C6_310721	Allele X:	GGATAATTTTCCCCAAAAAAGAAAAGTACT	GGGTTTGCAGCCGCTTCGTCAA
	Allele Y:	GATAATTTTCCCCAAAAAAGAAAAGTACC	
C8P19129409	Allele X:	CCCAAGCTACCTACAG	GTCTATTTAGTTCAGGTGATAAAGCTGCTT
	Allele Y:	CATGCTCCCAAGCTACCTACAC	
C9_12216080	Allele X:	CTGCTTGTATTATGGTTGTGCAGAT	CGTTTCTCAGCAGCTTTCTCAAAACATTT
	Allele Y:	CTGCTTGTATTATGGTTGTGCAGAC	
C9P27534079	Allele X:	GCAGCCACGAGTTTCCGGC	CTCAAAGTTCACAGTTGGAAGCTTCATT
	Allele Y:	GGCAGCCACGAGTTTCCGGT

**Table 2:** Information about molecular markers used for genotyping diploid gametes originated by tetraploid ‘Moncada’ mandarin as male and female parent, indicating accession number in Gene Bank or Phytozome, position in the reference clementine genetic map, noted alleles in ‘Moncada’ and reference

Marker	Gene bank/phytozome accession	Male–female parent	Marker type	Linkage group	Genetic position (cM)	Distance to centromere (cM)	Alleles	References
mCrCIR02G08	FR692362	M/F	SSR	1	16.73	43.93	244–246	Ollitrault et al. (2012a)
CIBE5720	ET082224	M	SSR	1	57.76	2.9	329–337	Ollitrault et al. (2010)
CIC2810-01	ET103213	F	SNP	1	63.40	2.74	AC	Ollitrault et al. (2012b)
EMA-M30	JX630064	F	SNP	1	69.72	9.06	CT	Garcia-Lor et al. (2013a)
CIC5950-02	ET083949	F	SNP	1	91.36	30.7	GA	Ollitrault et al. (2012b)
C1P26815936		M	SNP	1	117.56	56.9	TA	New
mCrCIR02D09	FR677569	M/F	SSR	2	13.37	43.53	236–238	Cuenca et al. (2011)
JK-CAC15	–	F	SSR	2	52.56	4.34	150–160	Kijas et al. (1997)
C2_23768463		M/F	SNP	2	81.04	24.14	GC	New
mCrCIR07D05	FR677574	M	SSR	2	90.41	33.51	185–189	Froelicher et al. (2008)
CIC3712-01	ET079481	F	SNP	2	93.92	37.02	CA	Ollitrault et al. (2012b)
JK-TAA41	–	M/F	SSR	2	160.74	103.84	154–163	Kijas et al. (1997)
MEST256	DY290355	F	SSR	3	17.02	73.58	209–225	García-Lor et al. (2012)
INVA-P855	JX630071	M	SNP	3	30.21	60.39	CT	Garcia-Lor et al.(2013a)
CIC4681-02	ET109640	F	SNP	3	92.78	2.18	TA	Ollitrault et al. (2012b)
C3_11509117		M/F	SNP	3	89.58	1.02	CG	New
CX0124	CN187496	M	SSR	3	110.27	19.67	164–170	In preparation
ATMR-M728	JX630073	F	SNP	3	141.92	51.32	GT	Garcia-Lor et al. (2013a)
CHS-M183	JX630074	M	SNP	3	167.33	76.73	GC	Garcia-Lor et al. (2013a)
C4P229604		M	SNP	4	0.802	15.29	GA	New
MEST070	DY268779	F	SSR	4	4.23	11.87	217–229	In preparation
CHI-M598	JX630074	F	SNP	4	11.37	4.73	GC	Garcia-Lor et al. (2013a)
C4P5278891		M	SNP	4	18.45	2.35	AG	New
mCrCIR06A02	AM489738	F	SSR	4	62.42	46.32	222–225	Froelicher et al. (2008)
C4P25377913		M	SNP	4	88.72	72.62	AG	New
CIC0446-01	ET091387	F	SNP	4	77.78	61.68	AT	Ollitrault et al. (2012b)
CI03D12a		M/F	SSR	4	90.06	73.96	261–281	Aleza et al. (2011)
MEST015	FC912829	M	SSR	5	16.21	6.89	174–186	García-Lor et al. (2012)
CMS30	–	M	SSR	5	31.35	8.25	150–152	Ahmad et al. (2003)
MEST104	DY273697	F	SSR	5	34.95	11.85	236–238	García-Lor et al. (2012)
CiC5842-02	ET083106	F	SNP	5	71.8	48.7	AC	Ollitrault et al. (2012b)
mCrCIR07E12	AM489750	M	SSR	5	95.43	72.33	138–142	Froelicher et al. (2008)
CiC2417-04	ET101382	F	SNP	5	103.36	80.26	TA	Ollitrault et al. (2012b)
C6_310721		M/F	SNP	6	0.32	5.88	TC	New
CIC2414-01	ET101372	F	SNP	6	8.11	1.91	AG	Garcia-Lor et al. (2013a)
C6_15847634		M	SNP	6	15.38	9.18	GT	New
LAPX-M238	JX630079	M/F	SNP	6	19.16	12.96	GC	Garcia-Lor et al. (2013a)
CI02F12	FR677570	F	SSR	6	60.84	54.64	122–130	Cuenca et al. (2011)
AOC-M290	JX630081	F	SNP	6	85.88	79.68	TC	Garcia-Lor et al. (2013a)
MEST123	DY276100	M	SSR	6	91.87	85.67	252–260	Aleza et al. (2011)
MEST107	DY274062	F	SSR	7	8.89	87.51	176–184	Cuenca et al. (2011)
FLS-M400	JX630083	M	SNP	7	45.99	50.41	CT	Garcia-Lor et al. (2013a)
mCrCIR03B07	FR677573	M/F	SSR	7	83.39	13.01	261–265	Cuenca et al. (2011)
CI07C07	AJ567409	M/F	SSR	7	98.01	1.61	227–234	Froelicher et al. (2008)
mCrCIR01F04a	AM489736	M/F	SSR	8	5.91	48.29	188–210	Froelicher et al. (2008)
CIC1208-01	ET070547	F	SNP	8	33.17	21.03	AG	Ollitrault et al. (2012b)
mCrCIR07B05	AM489747	F	SSR	8	57.78	3.58	203–209	Froelicher et al. (2008)
C8P19129409		M	SNP	8	77.07	22.87	CG	New
mCrCIR02C09	FR692359	F	SSR	8	95.32	41.12	248–255	Ollitrault et al. (2012b)
mCrCIR02A09	FR677568	M	SSR	8	98.18	43.98	152–162	Cuenca et al. (2011)
CIC5087-01	ET111514	F	SNP	9	15.88	36.32	TA	Ollitrault et al. (2012b)
C9_12216080		M/F	SNP	9	23.58	28.62	AG	New
mCrCIR07F11	FR677567	M/F	SSR	9	49.47	2.73	146–160	Kamiri et al. (2011)
C9P27534079		M	SNP	9	59.04	5.84	AG	New

*SSR* simple sequence repeat, *SNP* single nucleotide polymorphism, *M* male parent, *F* female parent

PCR amplifications using SSR markers were performed using a thermocycler rep gradient S (Eppendorf^®^) in 15 μL containing 0.5 μl 1U/μl of Taq DNA polymerase (Fermentas ^®^), 3 μL citrus DNA, 1.5 μl of 2 mM welled (Sigma ^®^) dye-labeled forward primer, 1.5 μl of 2 mM non-dye-labeled reverse primer, 0.2 mM of each dNTP, 1.5 μl 10× PCR buffer, and 0.45 μl 50 mM MgCl_2_. The PCR protocol was as follows: denaturation at 94 °C for 5 min followed by 40 cycles of 30 s at 94 °C, 30 s at 50 or 55 °C, and 30 s at 72 °C; and a final elongation step of 8 min at 72 °C. Capillary electrophoresis was carried out using a Genetic Analysis System 8000 (Beckman Coulter Inc.). The PCR products were initially denatured at 90 °C for 2 min, loaded at 2 kV for 30 s, and separated at 6 kV for 35 min. Alleles were sized based on a DNA size standard (400 bp). GenomeLab™ v.10.0 (Beckman Coulter Inc.) genetic analysis software was used for data collection.

SNP markers were genotyped using KASPar™ technology by LGC Genomics (Hoddesdon, UK). The KASPar™ genotyping system is a competitive, allele-specific dual Förster resonance energy transfer (FRET)-based assay for SNP genotyping. Primers were directly designed by LGC Genomics based on the SNP locus flanking sequence. Detailed explanation of the specific conditions and reagents used in KASPar™ technique can be found in Cuppen (2007). The allelic dose estimation in the heterozygous triploid hybrids was performed as described by Cuenca et al. (2013).

### Data analysis

#### Inferring the diploid gamete genetic configuration

In interploid crosses leading to triploid progenies, diploid gametes are transmitted from the tetraploid parent (Aleza et al. 2012a, b). For loci with completely different parental allelic configurations (A_1_A_2_ × A_3_A_4_), the genotype of the 2× gamete can be read directly from the configuration of triallelic triploid hybrids. When the female and male parents share one allele (A_1_A_2_ × A_2_A_2_ or A_1_A_2_ × A_2_A_3_), we inferred the structure of the 2× gamete forming biallelic triploid hybrids from the allelic dose, as described by Cuenca et al. (2011, 2013). We confirmed that all triploid hybrids were formed through the fusion of a diploid gamete from the tetraploid parent and a haploid gamete from the diploid parent by either observing triallelic configuration in the hybrids for at least one marker or from dosage estimation.

#### Parental heterozygosity restitution (PHR)

The PHR was calculated for each locus as the percentage of triploid individuals with the heterozygous allelic configuration inherited from tetraploid ‘Moncada’ mandarin transmitted through diploid gametes. Similarly, PHR was calculated for each individual as the percentage of loci with the same heterozygous allelic configuration as tetraploid ‘Moncada’ mandarin.

#### Estimation of preferential association frequency and maximum double reduction rate

For citrus, Stift et al. (2008) proposed a segregation model for allotetraploids, which was simplified by Aleza et al. (2016) for tetraploid resulting from somatic chromosome doubling. It is considered that in such tetraploid, for centromeric loci, the expected frequencies of each type of gamete depend only on the ‘tetrasomic’ parameter (τ), corresponding to the proportion of gametes formed by random associations of meiotic chromosomes (i.e., random bivalent or tetravalent pairing). The estimation of τ was performed using a maximum likelihood approach from the analysis of the marker closest to the centromere for each LG, as proposed by Aleza et al. (2016). This value ranges from 0 for completely disomic to 1 for complete tetrasomic inheritance. Confidence intervals (CIs) were estimated following a similar approach to the LOD drop-off method (Lander and Botstein 1989), by finding the values at either side of the estimated τ that corresponded to a tenfold decrease in probability. Then, preferential pairing (PP) was calculated as 1 − *τ*.

The double reduction rate (DR) and its confidence interval (CI) for each LG were estimated as proposed by Aleza et al. (2016). Briefly, DR is estimated from *τ* values for each LG for the markers furthest from the centromere applying a maximum likelihood approach, and the CI corresponds to the values on each side with a tenfold decrease in the probability.

#### Population diversity organization

Genetic differences between individuals were estimated using the DARwin6 software (Perrier and Jacquemound-Collet 2018) and analyzed with a neighbor-joining analysis using the simple matching dissimilarity index):

$$d_{i - j} = 1 - \frac{1}{L}\mathop \sum \limits_{l = 1}^{L} \frac{{m_{l} }}{\pi },$$

where $d_{i - j}$ is the dissimilarity between units *i* and *j*, *L* is the number of *loci*, $m_{l}$ is the number of matching alleles for *locus l,* and $\pi$ is the ploidy. From the dissimilarity matrix obtained, a weighted neighbor-joining tree (Saitou and Nei 1987) was computed.

The potential distortion in allelic segregation was analyzed using Chi-square test (*χ*^2^) with the Bonferroni correction for multiple testing applied (Bonferroni 1936; Goeman and Solari 2014; Holm 1979).

For group differentiation between the analyzed triploid hybrids of each progeny, the G/N relation was used, where G is the number of groups differentiated by the molecular markers used within each LG, and N is the total number of genotypes. The groups were obtained with the DARwin6 software (Perrier and Jacquemound-Collet 2018).

## Results and discussion

### Triploid genotyping

The genotyping of the triploid progenies was performed with 36 markers for MA and 31 for CM hybridizations, which allowed the unequivocal allelic differentiation between both parents and the determination of the origin of the diploid gametes that gave rise to each triploid hybrid.

**Fig. 1:**
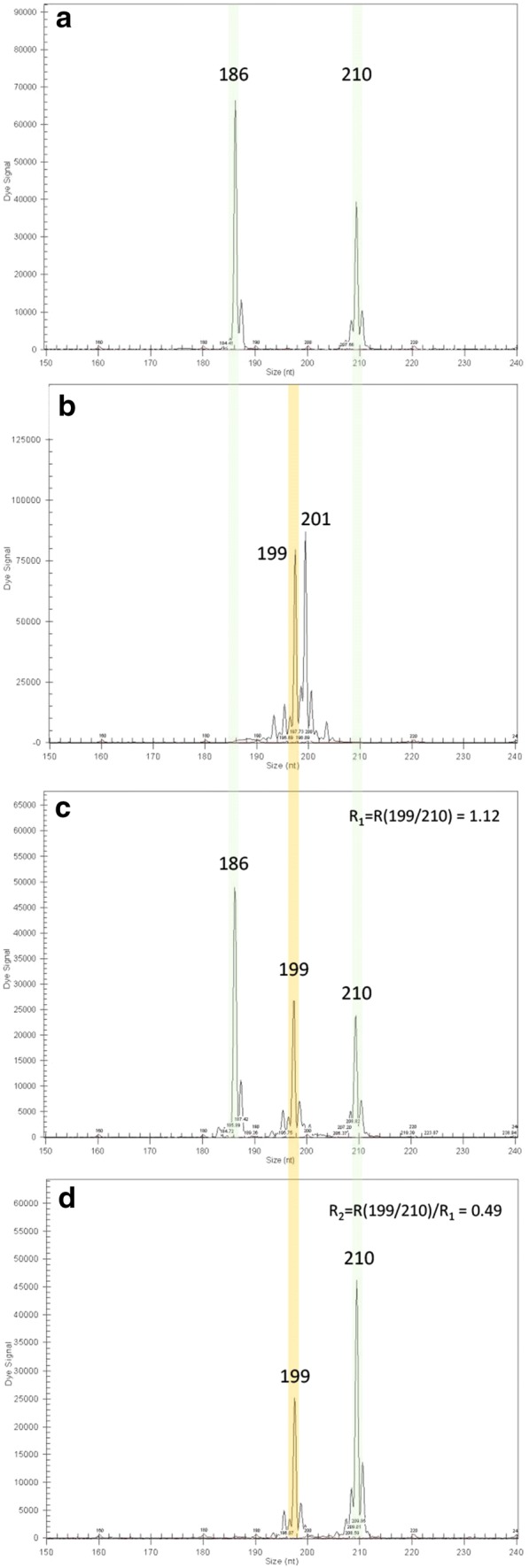
Electroferograms obtained using the CI01F04a SSR marker in: **a** tetraploid ‘Moncada’, **b** diploid ‘Anana’, **c** triallelic triploid hybrid MA14, **d** triploid hybrid MA50 with allelic dose correction using 199/210 ratio from MA14 as a baseline

Triallelic configurations with two alleles arising from tetraploid ‘Moncada’ were observed for all hybrids from MA for at least one SSR marker, directly confirming that the 2× gametes came from the tetraploid ‘Moncada’ progenitor. However, for CM hybrids, all molecular markers showed biallelic configurations, and the allele dosages were estimated as proposed by Cuenca et al. (2015). Finally, all triploid hybrids in both families were confirmed to arise from the fusion of a diploid gamete from tetraploid ‘Moncada’ and a haploid gamete from the diploid genitor (Fig. 1). Once the origin of the 2× gametes was confirmed, their genetic configurations were inferred for all marker-gamete combinations (Supplementary Table 1). An example for assessing genetic configuration from the direct observation of triallelic hybrids and the dosage estimation the peak ratio from a triallelic hybrid for the CI01F04a SSR marker is given in Fig. 1. In this case, tetraploid ‘Moncada’ shows 186/210 alelles (Fig. 1a) and ‘Anana’ 199/201 alleles (Fig. 1b). Hybrid ‘MA14’ shows 186/199/210 allele configuration (Fig. 1c), thus allows directly inferring 186/210 configuration for the 2× gamete from tetraploid ‘Moncada’ (heterozygosity restitution). In contrast, the hybrid ‘MA50’ for the same marker shows 199/210 allelic configuration (Fig. 1d), and therefore, the allelic dose estimation was done considering the relationship between the alleles 199/210 of the triallelic triploid hybrid as a baseline. It was concluded a 199/210:210 genotype for ‘MA50′ and consequently 210/210 genotypes for the 2× gamete from tetraploid ‘Moncada’ (no heterozygosity restitution).

The potential distortion in allelic segregation for the two types of homozygous gametes was analyzed using Chi-square test (*χ*^2^) with the Bonferroni correction for multiple testing applied. Only the marker MEST256 in LG3 for the MA population (Table 3) and the markers CHS-M183, MEST123 and FLS-M400 in LG3, LG6, and LG7, respectively, for the CM population presented distortion in allele segregations (Table 4).

**Table 3:** Estimation of parental heterozygosity restitution (PHR) frequency by diploid ‘Moncada’ ovules for each marker in triploid hybrids obtained from MA population and analysis of Mendelian allelic segregation (Chi-square test)

Locus	LG	Location	PHR	Chi square	*P* value
CI02G08	1	16.73	0.611	1.2857	0.257
CIC2810-01	1	63.40	0.569	0.0323	0.857
EMA-M30	1	69.72	0.569	0.0323	0.857
CIC5950	1	91.36	0.556	0.5000	0.480
Ci02D09	2	13.37	0.542	0.7576	0.384
CAC15	2	52.56	0.694	0.1818	0.670
C2_23768463	2	81.04	0.681	0.0435	0.835
CIC3712-01	2	93.92	0.542	0.7576	0.384
TAA41	2	160.74	0.542	6.8182	0.009
MEST256	3	17.02	0.542	22.0909	0.000
CIC4681-02	3	92.78	0.764	7.1176	0.008
C3_11509117	3	89.58	0.681	3.5217	0.061
ATMR-M728	3	141.92	0.625	6.2593	0.012
MEST70	4	4.23	0.764	4.7647	0.029
CHI-M598	4	11.37	0.833	0.0000	1.000
CI06A02	4	62.42	0.764	0.5294	0.467
CIC 0446-01	4	77.78	0.817	1.9231	0.166
CI03D12a	4	90.06	0.792	0.0667	0.796
MEST104	5	34.95	0.611	0.0000	1.000
CiC5842-02	5	71.8	0.528	0.0000	1.000
CiC2417-04	5	103.36	0.625	0.3333	0.564
C6_310721	6	0.32	0.597	0.0345	0.853
CICC2414-01	6	8.11	0.625	0.0370	0.847
LAPX-M238	6	19.16	0.611	0.1429	0.705
CI02F12	6	60.84	0.694	0.7273	0.394
AOC-M290	6	85.88	0.653	1.0000	0.317
MEST107	7	8.89	0.597	1.6897	0.194
CI03B07	7	83.39	0.583	0.5333	0.465
CI07C07	7	98.01	0.639	0.1538	0.695
CI01F04a	8	5.91	0.764	0.0588	0.808
CIC1208-01	8	33.17	0.792	0.0667	0.796
CI07B05	8	57.78	0.750	0.0000	1.000
CI02C09	8	95.32	0.653	0.3600	0.549
CIC5087-01	9	15.88	0.556	1.1250	0.289
C9_12216080	9	23.58	0.792	0.0667	0.796
CI07F11	9	49.47	0.583	0.5333	0.465

**Table 4:** Estimation of parental heterozygosity restitution (PHR) frequency by diploid ‘Moncada’ pollen for each marker in triploid hybrids recovered from CM population and analysis of Mendelian allelic segregation (Chi-square test)

Locus	LG	Location	PHR	Chi square	*P* value
CI02G08	1	16.73	0.614	2.941	0.086
CIBE5720	1	57.76	0.523	9.524	0.002
C1P26815936	1	117.56	0.636	0.500	0.480
CI02D09	2	13.37	0.557	0.231	0.631
C2_23768463	2	81.04	0.523	9.524	0.002
CI07D05	2	90.41	0.511	3.930	0.047
TAA41	2	160.74	0.432	0.080	0.777
INVAP855	3	30.21	0.648	1.581	0.209
C3_11509117	3	89.60	0.659	4.800	0.028
CX0124	3	110.27	0.500	0.818	0.366
CHSM183	3	167.33	0.466	11.255	0.001
C4P229604	4	0.80	0.750	1.636	0.201
C4P5278891	4	18.45	0.580	6.081	0.014
C4P25377913	4	88.72	0.568	6.737	0.009
Ci03D12a	4	90.06	0.557	7.410	0.006
MEST15	5	16.21	0.739	0.043	0.835
CMS30	5	31.35	0.500	1.455	0.228
CI07E12	5	95.43	0.534	5.488	0.019
C6_310721	6	0.30	0.705	0.154	0.695
C6_15847634	6	15.38	0.761	2.333	0.127
LAPXM238	6	19.16	0.667	0.034	0.853
MEST123	6	91.87	0.602	10.314	0.001
FLSM400	7	45.99	0.545	16.900	0.000
CI03B07	7	83.39	0.586	1.000	0.317
CI07C07	7	98.01	0.557	0.026	0.873
Ci01F04a	8	5.91	0.625	2.455	0.117
C8P19129409	8	77.07	0.568	8.526	0.004
Ci02A09	8	98.18	0.648	0.806	0.369
C9_12216080	9	23.58	0.724	0.000	1.000
CI07F11	9	49.47	0.602	2.314	0.128
C9P27534079	9	59.04	0.682	2.286	0.131

Other citrus studies showed segregation distortions. Bernet et al. (2010) analyzed reciprocal crosses between ´Fortune´ mandarin and ´Chandler´ pummelo, obtaining progenies with allelic frequencies distorted in both populations. In the same way, Ollitrault et al. (2012a) observed segregation distortions in male and female gametes of ´Clemenules’ clementine. In both studies, distortions were higher for the male gametes and the authors suggested that general factors such as mechanisms of gamete abortion, pollen competition, or gametophytic incompatibility could be related with them (Bernet et al. 2010; Ollitrault et al. 2012a).

### Genetic structure of diploid gamete populations arising from tetraploid ‘Moncada’ mandarin as female and male parent

#### Variability of PHR

**Fig. 2:**
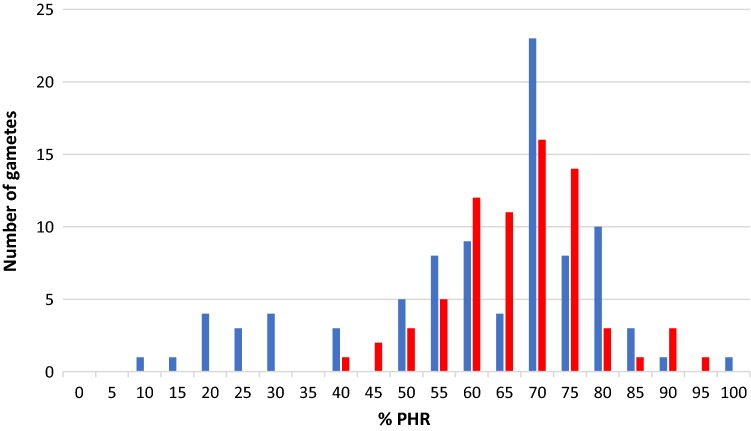
Distribution of PHR at the gamete level in the diploid gametes produced by tetraploid ‘Moncada’ mandarin used as female (red) or male parent (blue)

**Fig. 3:**
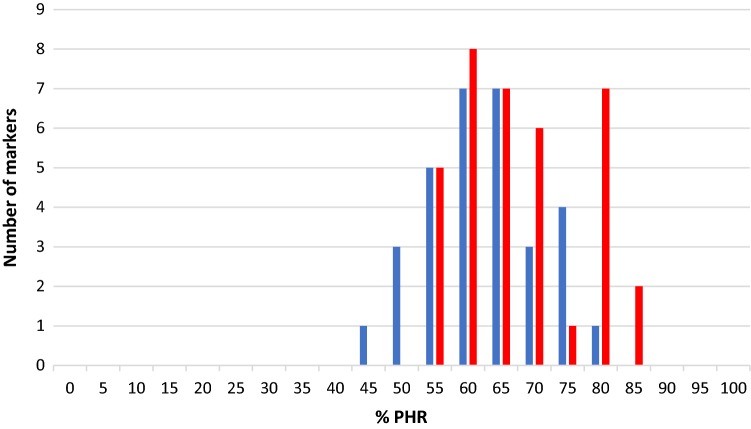
Distribution of PHR at the marker level in the diploid gametes originated by tetraploid ‘Moncada’ mandarin as female (red) and male parent (blue)

The PHR obtained from tetraploid ‘Moncada’ as male and female parent was calculated at gamete and marker level. At the gamete level, PHR presented a unimodal distribution when tetraploid ‘Moncada’ was used as female parent (Fig. 2), with a PHR average of 0.654 ± 0.093. The unimodal distribution observed in tetraploid ‘Moncada’ as female parent was similarly observed for tetraploid ‘Clemenules’ clementine analyzed by Aleza et al. (2016). In contrast, a more heterogeneous distribution was observed when used as male parent, displaying 14 diploid gametes (‘CM19’, ‘CM21’, ‘CM25’, ‘CM48’, ‘CM54’, ‘CM55’, ‘CM60’, ‘CM73’, ‘CM74’, ‘CM75’, ‘CM78’, ‘CM83’, ‘CM85’, and ‘CM86’) with very low PHR values, ranging from 0.10 to 0.40. Therefore, the average of PHR was a little bit lower (0.599 ± 0.085) (Fig. 2). At marker level, both populations displayed a unimodal distribution of PHR, although the diploid male gamete population showed lower PHR values, probably originated by the diploid male gametes with low PHR values (Fig. 3).

MA produced 2× gametes with PHR values ranging from 0.528 for the CIC5842-02 SNP locus in LG5 to 0.833 for the CHI-M598 SNP locus in LG4 (Table 3). For the remaining LGs, PHR values remain mostly constant along the chromosome. On the other hand, CM produced 2× gametes with PHR values ranging from 0.432 for the TAA41 SSR locus in LG2 to 0.761 for the C6_1584763 SNP locus in LG6 (Table 4).

Comparing tetraploid ‘Moncada’ as female and male parent, the largest differences are found in LG 4 and 8. As female parent, PHR values were 0.794 ± 0.31 for LG4 and 0.74 ± 0.06 for LG 8; as male parent, PHR values were 0.614 ± 0.091 and 0.614 ± 0.041 for LG4 and 8, respectively (Table 5).

**Table 5:** Estimation of the parental heterozygosity restitution for each LG, differentiated groups between genotypes analyzed and genetic distance in triploid populations arising from tetraploid ‘Moncada’ as male and female parent

Female gamete						Male gamete					
LG	PHR	PHR SD	*G*/*N*	Av D	Av D CI	LG	PHR	PHR SD	*G*/*N*	Av D	Av D CI
LG1	0.576	0.024	0.329	0.334	0.0087	LG1	0.591	0.060	0.213	0.3142	0.0059
LG2	0.600	0.080	0.548	0.313	0.0069	LG2	0.506	0.053	0.270	0.3632	0.0064
LG3	0.653	0.094	0.288	0.258	0.0065	LG3	0.568	0.099	0.270	0.3239	0.0058
LG4	0.794	0.031	0.288	0.182	0.0064	LG4	0.614	0.091	0.258	0.2966	0.0059
LG5	0.588	0.053	0.205	0.327	0.0087	LG5	0.591	0.129	0.236	0.3147	0.0060
LG6	0.636	0.039	0.301	0.297	0.0087	LG6	0.684	0.067	0.101	0.2601	0.0058
LG7	0.606	0.029	0.233	0.315	0.0084	LG7	0.563	0.021	0.213	0.3282	0.0066
LG8	0.740	0.060	0.329	0.225	0.0069	LG8	0.614	0.041	0.225	0.3029	0.0064
LG9	0.644	0.129	0.219	0.287	0.0074	LG9	0.669	0.062	0.112	0.2718	0.0059
TOTAL	0.654	0.093	1.000	0.278	0.0027	TOTAL	0.599	0.085	1.000	0.3086	0.0029

*LG* linkage group, *PHR* parental heterozygosity restitution, *PHR SD* standard deviation of the parental heterozygosity restitution, *G/N* number of genotypes on the total identified, *Av D* weighted average of the genetic distance, *Av D CI* confidence interval with *α* = 0.05 of genetic distance

#### Genotypic variability

**Fig. 4:**
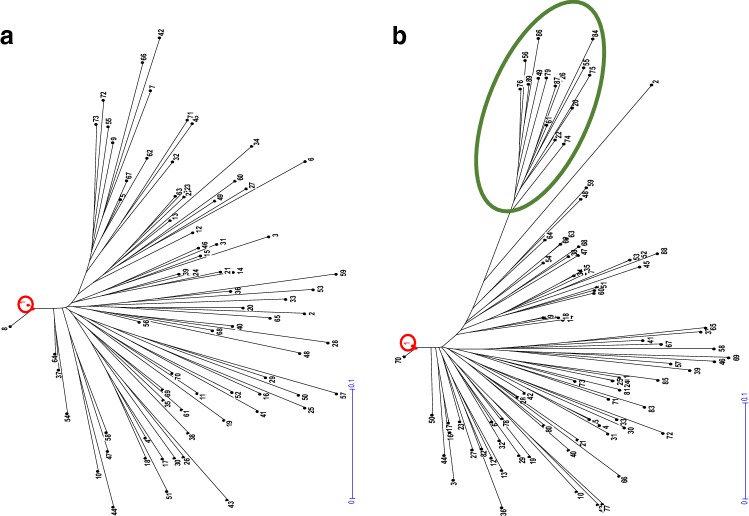
Dendrograms corresponding to the genetic analysis performed with SSR and SNP markers obtained by calculating the Simple Matching Dissimilarity Index and construction of the tree by weighted neighbor-joining of two populations of triploid hybrids regenerated from crosses **a** tetraploid ‘Moncada’ × ‘Anana’ and **b** ‘Clemenules’ × tetraploid ‘Moncada’. The red circle highlights the position of tetraploid ‘Moncada’. The green circle highlights the group of hybrids furthest from tetraploid ‘Moncada’

The genetic structure of these two populations was calculated by a neighbor-joining analysis (Fig. 4), allowing the differentiation of hybrid groups within each family and determine their genetic distance. The molecular markers used in this work made possible the differentiation of all triploid hybrids within each progeny (*G*/*N* = 1) (Table 5). The average genetic distance between gametes was slightly higher for CM (0.308 ± 0.0029) than for MA (0.278 ± 0.0027). In addition, the genetic structure of the MA population gametes is more homogeneous and compact than that obtained for the CM population. Comparing the genetic distances of both population gametes in relation to the tetraploid ‘Moncada’, CM displayed a genetic distance of 0.200 ± 0.093, whereas for MA, this distance was 0.173 ± 0.054. The results found for tetraploid ‘Moncada’, as male and female parent are consistent with those described by Aleza et al. (2016), which found a genetic distance value to tetraploid ‘Clemenules’ clementine of 0.176 ± 0.012 for the population of triploid hybrids obtained with this genotype as female parent. Nevertheless, in the CM gamete population, a group with higher genetic distance to the tetraploid ‘Moncada’ (0.362 ± 0.043) was observed (Fig. 4). This subpopulation is constituted by the same 14 diploid gametes described above with very low PHR. The genetic analysis performed in these hybrids reveals the same allele homozygosity configuration in nine (CIBE5720, C2_23768463, TAA41, CHSM183, C4P5278891, C4P25377913, Ci03D12a, Ci03B07, and C8P19129409) over the 31 molecular markers used, and also with two other SSR markers (MEST123 and Ci07D05) with the same homozygosity configuration except for only one diploid gamete. These molecular markers are located in all LGs, with the exception of LG9, and in the LG2 and LG6, three over the four markers analyzed in each LG, displayed the same allelic configuration in homozygosity.

### Preferential pairing (PP) and maximum double reduction (DR)

The genome of many cultivated citrus is composed of mosaics of the ancestral species (Curk et al. 2014, 2015; Wu et al. 2014, 2018). The works carried out on citrus phylogeny (Oueslati et al. 2017; Wu et al. 2014, 2018) have shown that the genomes of the progenitors that gave rise to ‘Moncada’ mandarin (‘Oroval’ clementine (*C. deliciosa* × *C. sinensis*) and ‘Kara’ mandarin (*C. unshiu* × *C. nobilis*) are constituted by an interspecific mandarin/pummelo mosaic structure; therefore, ‘Moncada’ mandarin also has an interspecific structure in its chromosomes.

*τ* and PP were calculated for each LG from the segregation data of the markers closest to the centromere using the probability models (Aleza et al. 2016). These markers were located between 1.0 and 24.1 cM from the centromere. For tetraploid ‘Moncada’ as female parent (Table 6), complete tetrasomic inheritance was the best model for seven out of the nine LGs (LG1, LG2, LG3, LG5, LG6, LG7, and LG9). For LG8, an intermediate inheritance with tendency towards a tetrasomic inheritance (PP = 0.375) was estimated, while the LG4 evidenced a clear intermediate inheritance (PP = 0.5). For tetraploid ‘Moncada’ as male parent (Table 7), most of the chromosomes fit the tetrasomic inheritance model with the markers used, with PP = 0 for LG1, LG2, LG3, LG4, LG7, LG8, and LG9, while LG5 and LG6 showed values that fit an intermediate inheritance model with tetrasomic tendency (PP = 0.215 and 0.115, respectively).

**Table 6:** Estimation of Preferential Pairing (PP) and Double Reduction (DR) rate for tetraploid ‘Moncada’ mandarin as female parent for markers located close and far from the centromere within each of the nine LGs

LG	Locus	DC (cM)	A_1_A_1_	A_1_A_2_	A_2_A_2_	τ	CI	PP	CI	DR	CI
1	CIC2810-01	2.7	16	41	15	1	1–0.845	0	0–0.165		
1	CI02G08	43.9	*11*	*44*	*17*			0		0.083	0–0.273
2	CAC15	4.3	10	50	12	0.915	1–0.595	0.085	0–0.195		
2	TAA41	103.8	*24*	*39*	*9*			0.085		0.251	0.05–0.458
3	C3_11509117	1.0	16	49	7	0.96	1–0.815	0.04	0–0.185		
3	MEST256	73.6	*30*	*39*	*3*			0.04		0.216	0.025–0.413
4	CHI–M598	4.7	6	60	6	0.5	0.790 – 0.260	0.5	0.180–0.740		
4	CI03D12a	74.0	*8*	*57*	*7*			0.5		0.125	0–0.467
5	MEST104	11.9	14	44	14	1	1–0.785	0	0–0.215		
5	CiC2417-04	80.3	*12*	*45*	*15*			0		0.063	0–0.251
6	CIC2414-01	1.9	13	45	14	1	1–0.805	0	0–0.195		
6	AOC-M290	79.7	*10*	*47*	*15*			0		0.021	0–0.208
7	CI07C07	1.6	14	46	12	1	1–0.735	0	0–0.265		
7	MEST107	87.5	*11*	*43*	*18*			0		0.104	0–0.293
8	CIC1208-01	3.58	7	57	8	0.625	0.965–0.355	0.375	0.035–0.645		
8	CI01F04a	48.3	*8*	*55*	*9*			0.375		0.067	0–0.347
9	CI07F11	2.7	13	42	17	1	1–0.820	0	0–0.180		
9	CIC5087-01	36.3	*13*	*40*	*19*			0		0.167	0–0.356

Allelic configurations for the loci used to estimate DR have been highlighted in italics

*LG* linkage group, *DC* distance to the centromere in cM [derived from reference genetic map data (Ollitrault et al. 2012a) and location of centromere (Aleza et al. 2015)], *A*_*1*_*A*_*1*_ number of individuals with that allelic configuration, *τ* tetrasomic rate, *CI* confidence interval, *PP* preferential pairing, *DR* double reduction rate

**Table 7:** Estimation of Preferential Pairing (PP) and Double Reduction (DR) rate for tetraploid ‘Moncada’ mandarin as male parent for markers located close and far from the centromere within each of the nine LGs

LG	Locus	DC (cM)	A_1_A_1_	A_1_A_2_	A_2_A_2_	τ	CI	PP	CI	DR	CI
1	CIBE5720	2.9	11	46	31	1	1–0.895	0	0–0.105		
1	C1P26815936	56.9	*14*	*56*	*18*			0		0.045	0–0.216
2	C2_23768463	24.1	11	46	31	1	1–0.895	0	0–0.105		
2	TAA41	103.8	*26*	*38*	*24*			0		0.352	0.181–0.518
3	C3_11509117	1.0	21	58	9	1	1–0.715	0	0–0.285		
3	CHSM183	167.3	*12*	*41*	*35*			0		0.301	0.130–0.468
4	C4P5278891	2.4	11	51	26	1	1–0.850	0	0–0.150		
4	Ci03D12a	74.0	*28*	*49*	*11*			0		0.165	0–0.336
5	MEST15	6.9	12	65	11	0.785	1–0.510	0.215	0–0.490		
5	CI07E12	72.3	*13*	*47*	*28*			0.215		0.390	0.174–0.608
6	C6_310721	5.9	14	62	12	0.885	0.96–0.815	0.115	0.04–0.185		
6	MEST123	85.7	*27*	*53*	*8*			0.115		0.174	0–0.368
7	CI07C07	1.6	19	49	20	1	1–0.870	0	0–0.130		
7	FLSM400	50.4	*33*	*48*	*7*			0		0.182	0.015–0.353
8	C8P19129409	22.9	28	50	10	1	1–0.850	0	0–0.150		
8	Ci01F04a	48.3	*21*	*55*	*12*			0		0.063	0–0.234
9	CI07F11	2.7	22	53	13	1	1–0.825	0	0–0.175		
9	C9_12216080	28.6	*12*	*63*	*12*			0		0.000	0–0.102

Allelic configurations for the loci used to estimate DR have been highlighted in italics

*LG* linkage group, *DC* distance to the centromere in cM [derived from reference genetic map data (Ollitrault et al. 2012a) and location of centromere (Aleza et al. 2015)], *A*_*1*_*A*_*1*_ number of individuals with that allelic configuration, *τ* tetrasomic rate, *CI* confidence interval, *PP* preferential pairing, *DR* double reduction rate

Likewise, clementines also present an interspecific mandarin/pummelo structure (Wu et al. 2018) Aleza et al. (2016) studied the segregation model in tetraploid ‘Clemenules’ clementine as female parent, obtaining very similar results, as we report for the tetraploid ‘Moncada’ mandarin, generally fitting the tetrasomic inheritance model except for LG4, which fitted the intermediate inheritance model. However, they also reported that the LG6 and LG8 showed values that fit the intermediate inheritance model, with high tetrasomic tendency. Comparatively, we found that for ‘Moncada’ mandarin as female parent, the LG6 shows tetrasomic segregation, while results for the LG8 agree with the after as was reported for the tetraploid ‘Clemenules’ clementine, but with higher PP value. Subsequently, Rouiss et al. (2018) analyzed the segregation model of the tetraploid ‘Mexican’ lime (*C. aurantiifolia*), which originated from an interspecific hybridization between *C. micrantha* (papeda) and *C. medica* (Citron) (Curk et al. 2016; Nicolosi et al. 2000; Wu et al. 2018). The results showed that tetraploid ‘Mexican’ lime has intermediary inheritance with a preferential disomic trend. In addition, Kamiri et al. (2018) assessed the meiotic behavior of an intergeneric tetraploid somatic hybrid resulting from symmetric protoplast fusion of diploid *C. reticulata* and diploid *Poncirus trifoliata*, and observed an intermediate inheritance with a preferential disomic trend. On the other hand, the genotyping of the triploid progeny derived from a cross between diploid pummelo (*C. maxima*) and an allotetraploid intergeneric somatic hybrid between *C. reticulata* and *C. limon* showed a tetrasomic and intermediate inheritance for this citrus interspecific somatic hybrid (Kamiri et al. 2011). Altogether, these studies reveal that the preferential pairing of tetraploid citrus genotypes greatly varies in relation to their constitutive genomes. The differentiation between *C. medica* and *C. micrantha* as well as the one between *C. reticulata* and *P. trifoliata* seems to have a much more impact in preferential pairing than the one between *C. maxima* and *C. reticulata*. Tetraploid ´Moncada´ differs slightly in the segregation model when used as female or male parent. These sex-specific differences were also observed for salmon fish (Allendorf and Danzmann 1997). Disomic segregation was observed in females, while segregation in males was best explained by a mixture of disomic and tetrasomic inheritance.

The tetraploid ´Moncada´ as female parent showed significant values of DR in LG2, LG3, LG4, LG7, and LG9. For all LGs, the confidence intervals (CI) for DR values include the value of 1/6, considered as the maximum value of DR for tetrasomic segregation and one crossover event occurring between the marker and the corresponding centromere (Haynes and Douches 1993; Mather 1936; Bourke et al. 2015), although LGs 2 and 3 displayed a higher estimation of DR. When tetraploid ‘Moncada’ was used as male parent, significant values of DR were obtained for LG2, LG3, LG4, LG5, LG6, and LG7. For LG3, LG4, LG6, and LG7, the confidence intervals (CI) for DR values include the maximum value of DR under the hypothesis described above. In addition, LG2 and LG5 showed higher DR values. Tetraploid ‘Moncada´ shows the same trend as female and male parent in DR values for LG1, LG2, LG3, LG4, LG7, and LG8. The frequency of DR considers maximum values of 0 for random chromosome segregation hypothesis, 1/7 with pure random chromatid segregation hypothesis, and 1/6 with complete equational segregation (Mather 1935; Muller 1914). Estimated values over 1/6 should be due to the segregation distortion observed for the corresponding markers. Indeed, our model analysis is based on Mendelian segregation hypothesis, while negative sporophytic selection for dominant gene may induce a diminution of heterozygous frequencies (for the gene and linked markers) and results in overestimation of DR. Different works have been performed with the objective to estimate the DR frequency and these values have been ranged from 0 to almost 0.30 (Fisher 1947, 1950; Haynes and Douches 1993; Tai 1982a, b; Welch 1960; Wu et al. 2001). The values of DR rate can differ between loci according the tetrasomic inheritance model. This variability depends on both the chromosome in which the marker is located and the position of the marker within the chromosome. There are chromosomes with a greater tendency to form multivalent that would originate higher values of DR (Butruille and Boiteux 2000). In addition, DR could be better estimated using larger populations (Butruille and Boiteux 2000) and it is more probable to occur in markers located in telomeric rather than in centromeric regions, in which the probability of recombination events is close to zero (Aleza et al. 2015; Butruille and Boiteux 2000; Welch 1960). In addition, Butruille and Boiteux (2000) indicated that DR causes a decrease of the equilibrium frequencies of deleterious alleles, and it has much more influence on genes subjected to gametophytic selection than on genes solely under sporophytic selection. With gametophytic selection, low frequencies of DR are enough to reduce equilibrium frequencies several folds.

### Implications for citrus-breeding programs

Two strategies are routinely exploited for obtaining citrus triploids, i.e., interploid hybridizations between 2× and 4× parents (Aleza et al. 2012a, b; Starrantino and Recupero 1982) and through female 2n gametes (Aleza et al. 2010; Cuenca et al. 2011, 2015). In interploid hybridizations, the tetraploid parent results usually from somatic chromosome doubling arising spontaneously in nucellar cells or induced by colchicine treatment. The study of the origin of the diploid gametes, which greatly influences the structure of the resulting triploid hybrid populations, is of great interest to select the most appropriate strategies to obtain new hybrids with desired characteristics. Cuenca et al. (2015) demonstrated that SDR mechanism gives rise to the 2n megagametophytes in diploid ‘Moncada’ mandarin. The use of this strategy produces hybrid progenies with large genetic variation, due to the relatively low transmission of the parental heterozygosity to the offspring (about 40% on average), thus resulting in high number of new allelic multilocus combinations. In this paper, we have analyzed the chromosome segregation in the tetraploid ‘Moncada’ mandarin, which showed predominantly tetrasomic segregation, when used both as female and male parent, with an average PHR of 65% when used as female and 60% as male parent. Moreover, PHR is relatively constant along the chromosomes. Therefore, if we compared with SDR-2n female gametes, interploid hybridizations with tetraploid ‘Moncada’ mandarin as tetraploid parent are potentially a more efficient strategy for the development of new varieties that are genotypically more similar to the ‘Moncada’ mandarin.

Furthermore, depending on the LG in which a gene controlling an eventual trait of interest is located, the genetic regulation of the trait and the direction of the crossing, different segregation in the offspring can be obtained. For example, the PHR in LG8 is higher when tetraploid Moncada is used as female than as male parent, and therefore, the progeny will show higher heterogeneity in this LG when using tetraploid Moncada as male parent. Considering a trait of interest controlled by a single dominant allele at a locus in LG8, the probability to obtain triploid hybrids that inherit the trait of interest is higher using tetraploid ‘Moncada’ as female parent.

Tetraploid ´Moncada´ mandarin displayed significant values of DR as male and female parent. DR results in a decrease of PHR and thus an increase of inbreeding (Haynes and Douches 1993). The production of higher levels of homozygosity could be useful in triploid mandarin breeding for the potential cleaning effect that DR can have by revealing deleterious alleles to selection (Butruille and Boiteux 2000; Bourke et al. 2015). DR also could increase the accumulation of rare but favorable allelic configurations through selection with molecular markers (Bourke et al. 2015).

The knowledge of the difference in segregations according to the crossing strategy (2n gametes or interploid hybridization) to obtain hybrid triploid progenies with the ‘Moncada’ mandarin opens a range of possibilities for designing efficient breeding programs aimed to obtain innovative products to fulfill the market demands.

## Conclusions

The analysis of codominant marker segregation over the nine citrus chromosomes allowed to unravel the segregation pattern of the tetraploid ‘Moncada’. Using both as female and male parent, it displayed tetrasomic inheritance for most LGs, with slight variations according to the direction of the crossing. As female parent, LG8 showed intermediate inheritance with tendency towards tetrasomic inheritance, and LG4 evidenced clear intermediate inheritance. As male parent, LG5 and LG6 showed values that fit an intermediate inheritance model with tetrasomic tendency. Significant DR rates were found in LG2, LG3, LG4, LG7, and LG9 when using tetraploid Moncada as female parent and in LG2, LG3, LG4, LG5, LG6, and LG7 as male parent. Likewise, differences in PHR were found between tetraploid ‘Moncada’ as female parent and male parent, with higher values in LG 4 and LG 8 as female parent. The new knowledge generated here will serve to define crossing strategies in citrus improvement programs to efficiently obtain new varieties of interest in the global fresh consumption market.

## Electronic supplementary material

Below is the link to the electronic supplementary material.

Supplementary material 1 (XLSX 1278 kb)
